# A randomised controlled trial to assess the effectiveness of offering study results as an incentive to increase response rates to postal questionnaires [ISRCTN26118436]

**DOI:** 10.1186/1471-2288-5-34

**Published:** 2005-10-26

**Authors:** Sarah Cockayne, David J Torgerson

**Affiliations:** 1 University of York, York Trials Unit, Department of Health Sciences, Area 4 Seebohm Rowntree Building, York, UK

## Abstract

**Background:**

Postal questionnaires are widely used to collect outcome data on participants. However, a poor response to questionnaires will reduce the statistical power of the study and may introduce bias. A meta analysis of ten trials offering study results, largely in the fields of education and marketing, was shown to be ineffective, with the odds ratio for response with offering research findings is 0.92 (95% CI 0.75 to 1.11). However uncertainty still exists as it is uncertain whether results from such trials can be extrapolated to that of a health care setting. The aim of this study was to assess whether offering participants study results increases the response rates to postal questionnaires.

**Methods:**

1038 women aged over 70 years were remotely randomised by computer in a 3:1 ratio. 250 participants did not receive the offer of knowing the results of the trial and 788 participants were offered the results of the trial in a postal questionnaire. The main outcome measure was response rate. Chi square test was used to evaluate the overall differences in response rate between the two groups. An adjusted analysis, adjusting for whether the participant was taking calcium and age was also undertaken.

**Results:**

The response rates were not significantly different Odds Ratio 0.88 (95% confidence intervals 0.48 to 1.63) p = 0.69.

**Conclusion:**

Offering study results to women living in the community aged over 70 does not increase response rates to postal questionnaires. Although researchers have an ethical obligation to offer participants study results, since 10% of women did not wish to receive the results, investigators should give participants the option to opt out of receiving the study's results.

## Background

Postal questionnaires are widely used to collect data on participants in health research. They are an attractive means of collecting data, because they are easy to administer and may be the only economically viable method of collecting data on large numbers of participants who may be geographically dispersed. However, if there is a poor response rate to these questionnaires the validity of the study may be at risk for two main reasons. First, the statistical power of the study will be reduced. Second, bias may be introduced if non-responders differ significantly from those who do respond. In other words low response can threaten the internal validity of randomised trials as bias can be introduced and the external validity of surveys are undermined as non-response will prevent random sampling. Identifying effective strategies to increase response rates to postal questionnaires is therefore highly relevant to researchers since it could improve the quality of their research.

Offering participants the chance of being informed of the results of the study in which they are participating could improve response rates for three reasons. First it demonstrates the researcher's goodwill and sincerity [1,2]. Second, it adds greater credibility and importance to the respondent's efforts and third, it could act as a non-monetary incentive. In 2002 Edwards [3] identified 75 different strategies for increasing response rates including non-monetary incentives versus no incentive, which were shown to be effective (odds ratio of 1.19 95% confidence interval 1.11 to 1.28). In 2004 the same review group reported the results of a meta analysis of ten trials offering study results as an incentive which included 13 642 participants [4]. When the results of these trials were pooled in a random effects meta analysis the odds ratio for response with research findings was 0.92 (95% CI 0.75 to 1.11). However, uncertainty still exists as to the effectiveness of this strategy in a health care setting. This is because only one of these trials [5] was undertaken in a health care setting. The results of this study showed no evidence to suggest that promising study results to participants increases response rates. However, participants were hospital chief executives who were sent questionnaires regarding managerial issues such as expenditure and not items directly relating to their health. The effectiveness of this strategy has yet to be tested in a setting related directly to the population's health and it is uncertain whether trials conducted in other areas such as marketing and education can be extrapolated to a health care setting. The aim of this study therefore was to assess whether offering to tell participants the results of the study would increase the response rates to postal questionnaires in a health care setting

## Methods

### Study population

The subjects in this study were community dwelling women aged over 70 living in the York and Cumbria area. These women were due to be sent a final follow-up questionnaire as part of a multi-centred randomised controlled trial of calcium and vitamin D supplementation for fracture prevention. Participants had been originally assigned to one of two groups. The intervention group received daily oral supplementation of 1,000 mg of calcium with 800 IU vitamin D_3 _with a patient information leaflet on dietary calcium intake and falls prevention. The control group received the patient information leaflet only. Further details of this trial have been reported elsewhere [6].

### Inclusion/exclusion criteria and randomisation

Women were eligible for this trial if they had been recruited from the York centre and were due to receive their final follow-up questionnaire in March 2004. Women were excluded if they had withdrawn from the trial or if notification of the participant's death had been received. An independent researcher from the York Trials Unit randomised eligible women in a 3:1 ratio in favour of offering the results of the trial, by computer. An unequal randomisation ratio was used for two reasons. First, there was an issue of administrative convenience. The 'standard' letter to trial participants was to offer them the study results and this letter was going out to all centres including those not participating in this study. Therefore, it was less costly with lower administrative input to produce a smaller separate batch of letters to send out than if we had used equal allocation. Second, although we had some uncertainty with respect to the direction of the effect (hence the trial) on balance we expected that the response rates would be lower in the group assigned to 'not offered the results.' If this had been the case and more participants were assigned to this group, then the overall response rate to the final follow-up would have been reduced.

### Control

250 women assigned to the group 'not offered the results' of the trial received a one-page questionnaire asking whether the participant had had a fracture in the past three years, how much calcium and vitamin D supplement they were taking and if applicable the reason for stopping the supplement, along with a request to give permission to send details of future research. However, all participants returning their questionnaire would be informed of the trial's results.

### Intervention

788 women assigned to 'offered the results' received the same questionnaire but with an additional question asking whether they would like to be notified of the results of the trial.

Both groups received a personalised cover letter showing university sponsorship, along with a business reply envelope. Those participants not returning a questionnaire within three weeks were sent up to two reminder letters, questionnaires and business reply envelopes, three and six weeks after the initial mailing. Administration of the questionnaire was not blind to group allocation.

Ethical approval for the study was obtained from the Northern and Yorkshire multicentre research ethics committee and relevant local research ethics committees.

### Outcome

The primary outcome was return of final follow-up questionnaire or reminder by the participant.

### Sample size and statistical analysis

The sample size for this study was calculated to give 95% power (5% two sided significance) to detect a 10% difference in response rates. We assumed that the baseline response rate would be approximately 80%, a figure based on work from a previous trial conducted by the authors on women of a similar age and risk of fracture [7]. Results were analysed using SPSS 11. All participants were included in the analysis (intention to treat). We used Chi square test to evaluate the overall differences in response rate between the two groups. We also undertook an adjusted analysis, adjusting for whether the participant was taking calcium and age. Statistical analysis was not undertaken blind to group allocation.

## Results

**Figure 1:**
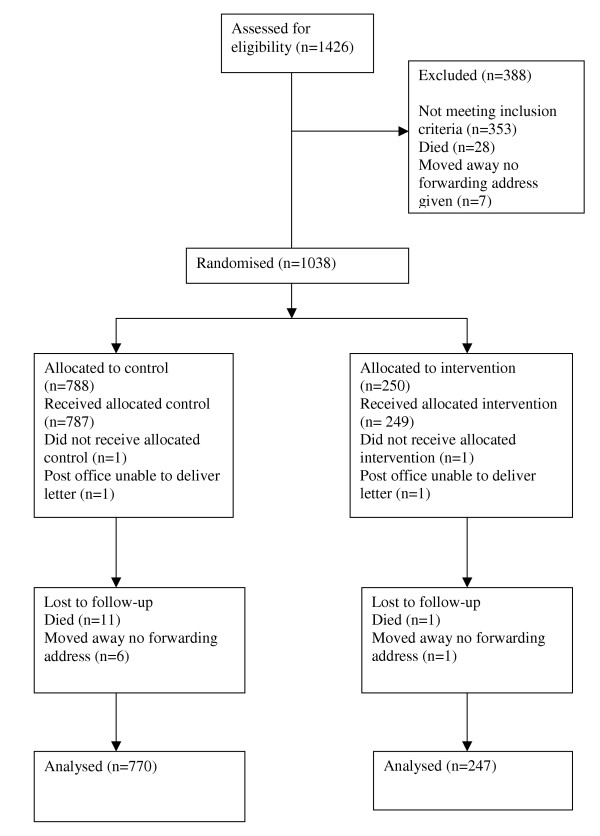
Origin and flow of participants in trial.

The baseline characteristics for the participants are shown in table 1 whilst figure 1 describes the participant recruitment profile. The overall response rates are shown in table 2. A total of 153 participants were sent reminder questionnaires,112 of which were in the intervention group and 41 in the control group. 63 participants (56%) in the intervention group and 27 participants (66%) in the control group returned a reminder. These response rates were not significantly different. Chi square = 0.16 df = 1 p = 0.69 Odds Ratio (OR) 0.88 (95% Confidence Intervals [CI] 0.48 to 1.63). As the study was a factorial design, the data were tested to ensure there was no interaction between taking calcium and being allocated to being either offered/not offered the results of the trial. Adjusted OR for returning the postal questionnaire showed no association between offering the results of the trial and the return of the questionnaire (OR 0.81 [CI 0.44 to 1.51] p = 0.51) whilst taking calcium significantly predicted (OR 5.10 [CI 2.02 to 12.84] p = 0.001) the return of the questionnaire as did increasing age, but to a lesser degree (OR 1.05 [CI 1.00 to 1.11] p = 0.05).

**Table 1:** Baseline characteristics of participants

**Baseline Variable**	**Intervention**	**Control**
Age (mean, SD)	76.2 (4.41)	77.09 (5.34)
Weight (Kilos, mean SD)	65.23 (11.88)	65.65 (12.45)
Percent (n) < 58 kilos	31.2% (231/740)	30.8% (72/234)
Prior any fracture	59.0% (454/770)	61.5% (152/247)
Smoker	5.8% (32/551)	5.4% (9/166)
Poor/Fair Health	33.8% (260/769)	28.7% (71/247)
Maternal hip fracture	13.6% (105/770)	15.0% (37/247)
Fall in previous 12 months	31.6% (243/770)	36.8% (91/247)
Taking anti-fracture treatment	5.5% (42/770)	3.2% (8/247)
SF12		
MCS (mean; SD)	52.20 (9.05)	53.00 (8.66)
PCS (mean; SD)	41.33 (11.88)	40.28 (11.60)
Euroqol	0.73 (0.218)	0.69(0.26)

**Table 2:** Response rate to final follow up questionnaire

	**Intervention**	**Control**
Replied	721	233
Not replied	49	14
Total number of questionnaires analysed	770	247
Overall response rate	93.6%	94.3%

Of the 721 participants returning questionnaires in the group offered the results of the trial, 647 (89.7.%) requested the results of the study and three women in the group 'not offered the results' pro-actively asked to be sent a copy of the results. The baseline characteristics of participants who did and did not request the study's results are reported in table 3. Adjusted OR showed taking calcium (OR 4.00 [CI 1.88 to 8.51]) p < 0.05) significantly predicted whether participants requested trial results, however age (OR 0.96 [CI 0.91 to 1.01]) p = 0.12) and fair or poor health (OR 0.66 [CI 0.40 to 1.10]) p = 0.11) did not predict whether participants requested trial results.

**Table 3:** Baseline characteristics of participants who did and did not request the trial's results

**Baseline Variable**	**Requested trial results**	**Did not request trial results**
Age (mean, SD)	76.00 (4.30) n = 647	76.80 (4.68) n = 74
Weight (Kilos, mean SD)	65.59 (11.94) n = 618	64.28 (11.77) (n = 71)
Percent (n) < 58 kilos	30.7% (190/618)	32.4% (23/71)
Prior any fracture	59.2% (383/647)	53.4% (39/73)
Smoker	4.5% (21/463)	3.8% (2/52)
Poor/Fair Health	31.9% (206/646)	41.7% (30/72)
Maternal hip fracture	13.3% (86/647)	16.7% (12/72)
Fall in previous 12 months	31.1% (201/647)	31.9% (23/72)
Taking anti-fracture treatment	5.9% (38/647)	4.2% (3/73)
SF12		
MCS (mean; SD)	52.61 (8.49) n = 629	50.98 (11.5) n = 70
PCS (mean; SD)	41.73 (11.8) n = 629	39.65 (11.20) n = 70
Euroqol	0.74 (0.21) n = 640	0.75 (0.19) n = 71

The characteristics of those who did and did not respond to the questionnaire are reported in table 4.

**Table 4:** Baseline characteristics of responders and non-responders

**Variable**	**Responders**	**Non-responders**	**p value**
Age (mean, SD)	76.32 (4.62) n = 954	77.43 (5.22) n = 63	0.07
Weight (Kilos, mean SD)	65.54 (12.06) n = 912	62.29 (10.93)	0.04
Percent (n) < 58 kilos	30.8% (281/912)	35.5% (22/62)	0.02
Prior any fracture to randomisation	59.4% (567/954)	61.9% (39/63)	0.80
Smoker	4.5% (30/671)	23.9% (11/46)	< 0.05
Poor/Fair Health	31.7% (302/953)	46.0% (29/63)	0.03
Maternal hip fracture	14.0% (134/954)	12.7% (8/63)	0.91
Falls in 12 month prior to randomisation	32.3% (308/954)	41.3% (26/63)	0.18
Taking anti-fracture treatment	5.0% (48/954)	3.2% (2/63)	0.72
SF12			
MCS (mean; SD)	52.63 (8.78) n = 929	48.53 (10.6) n = 60	0.01
PCS (mean; SD)	41.29 (11.68) n = 929	37.66 (13.36) n = 60	0.02
Euroqol	0.73 (0.22) n = 938	0.62 (0.30) n = 62	0.01

## Discussion

This study found no evidence that offering study results to participants increased the response rate to a postal questionnaire. There are two possible reasons for this strategy being ineffective. First, offering results of the trial may not act as an incentive to participants to respond because it appeals only to those interested in the trial and who were likely to respond anyway. If this were the case then offering the results of the trial was insufficient incentive to motivate non-responders. Second, the following strategies were adopted which are known to increase response rate; a short, one-sided, straightforward questionnaire designed to be of interest to participants but not containing sensitive questions, reminders with a second copy of the questionnaire and university sponsorship. This combination of strategies produced a high response rate of over 93% and it may be that any strategy would struggle to significantly increase the response rate further (i.e., a ceiling effect).

This study was undertaken among women over 70 living independently in the community therefore it is not possible to generalise these results to either men, women younger than 70 or to those living in nursing homes or other forms of residential care. Also because the participants had already agreed to take part in a randomised controlled trial the results may not apply to the wider population of women over 70 years. The incentive of 'offering trial results' was tested in combination with other strategies known to increase response rates, which together produced an excellent response rate, which any strategy would find difficult to improve upon. It may be that this incentive is effective in a different combination of strategies.

## Conclusion

Although offering the results of the study did not increase the response rates, researchers do have an ethical obligation to offer participants the results of study if they wish to receive them. Our results show that 90% of women did want to know the results of our trial however, 10% did not wish to receive the results, which emphasises the need for investigators to give participants the option to 'opt out of' receiving the trial's results. Within the UK, for new studies the issue of disseminating study results will have to be addressed since the introduction of the new Research Ethics Committee (REC) form in 2004. The investigator is asked whether the study's results will be disseminated to participants and details of what will happen to the results of the research, when the results are likely to be published and how participants can obtain a copy of the results needs to be stated on the patient information leaflet.

## Competing interests

Professor David Torgerson Director of the York Trials Unit (PhD) has received funding from SHIRE and other pharmaceutical companies for research and sponsorship to attend conferences and meetings.

## Authors' contributions

SC was a trial coordinator and was responsible for data entry and cleaning, combining the datasets, undertaking the analysis and wrote the first draft of the report.

DJT principal investigator, drafted the trial protocol and helped obtain funding for the main study, helped to draft the manuscript and is guarantor for the paper.

## Pre-publication history

The pre-publication history for this paper can be accessed here:


